# Structure-Based Mechanism for Early PLP-Mediated Steps of Rabbit Cytosolic Serine Hydroxymethyltransferase Reaction

**DOI:** 10.1155/2013/458571

**Published:** 2013-07-15

**Authors:** Martino L. Di Salvo, J. Neel Scarsdale, Galina Kazanina, Roberto Contestabile, Verne Schirch, H. Tonie Wright

**Affiliations:** ^1^Dipartimento di Scienze Biochimiche, Sapienza Università di Roma, 00185 Roma, Italy; ^2^Center for the Study of Biological Complexity and Institute for Structural Biology and Drug Discovery, Richmond, VA 23284-2030, USA; ^3^Department of Biochemistry and Institute of Structural Biology and Drug Discovery, Virginia Commonwealth University, Richmond, VA 23219, USA

## Abstract

Serine hydroxymethyltransferase catalyzes the reversible interconversion of L-serine and glycine with transfer of one-carbon groups to and from tetrahydrofolate. Active site residue Thr254 is known to be involved in the transaldimination reaction, a crucial step in the catalytic mechanism of all pyridoxal 5′-phosphate- (PLP-) dependent enzymes, which determines binding of substrates and release of products. In order to better understand the role of Thr254, we have expressed, characterized, and determined the crystal structures of rabbit cytosolic serine hydroxymethyltransferase T254A and T254C mutant forms, in the absence and presence of substrates. These mutants accumulate a kinetically stable *gem*-diamine intermediate, and their crystal structures show differences in the active site with respect to wild type. The kinetic and crystallographic data acquired with mutant enzymes permit us to infer that conversion of *gem*-diamine to external aldimine is significantly slowed because intermediates are trapped into an anomalous position by a misorientation of the PLP ring, and a new energy barrier hampers the transaldimination reaction. This barrier likely arises from the loss of the stabilizing hydrogen bond between the hydroxymethyl group of Thr254 and the **ε**-amino group of active site Lys257, which stabilizes the external aldimine intermediate in wild type SHMTs.

## 1. Introduction

 Serine hydroxymethyltransferase (SHMT: EC 2.1.2.1) is a ubiquitous pyridoxal 5′-phosphate- (PLP-) dependent enzyme that catalyzes the reversible interconversion of L-serine and glycine, coupled to the formation and breakdown of 5,10-methylenetetrahydrofolate (5,10-CH_2_-H_4_PteGlu) [1, 2]. Because of its essential role in one-carbon units metabolism, SHMT has been often indicated as a potential target of chemotherapeutic agents [3–5]. It also catalyzes the conversion of 5,10-methenylene- to 5-formyl-H_4_PteGlu [6], the transamination and racemization of D- and L-alanine [7], the retro-aldol cleavage of *erythro* and *threo* isomers of both L-threonine and L-*β*-phenylserine [8], and the decarboxylation of aminomalonate [9]. PLP-dependent enzymes exist in complexes that absorbs in the 310 nm to 500 nm range as the result of a conjugated *π*-electron system. These absorption properties have played an important role in elucidating the mechanisms of PLP addition and catalysis, since several intermediates on the reaction pathway have unique structural and absorbance characteristics [10, 11]. SHMT is distinctive among the PLP-dependent enzymes in the number of these absorbing complexes that can be observed, and these have been exploited to determinate kinetic rates for their interconversion by stopped-flow and temperature jump spectroscopy [11–13].

**Scheme 1:**
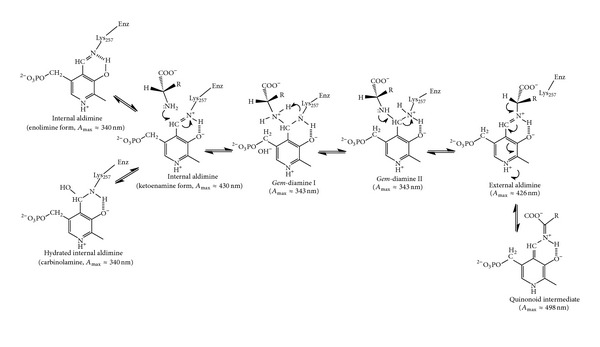
Mechanism of transaldimination reaction of SHMT.

 In PLP-dependent enzymes, the 4′-aldehyde of PLP is bound as an aldimine to the *ε*-amino moiety of an active site Lys residue in what is called the “internal aldimine.” For those PLP-dependent enzymes that catalyze reactions involving substrate amino acids, the initial step in the catalytic reaction is the formation of the “geminal diamine” (*gem*-diamine) between the C4′ aldehyde of PLP and the amino group of the substrate. The orientation of the substrate in the active site with respect to the plane of PLP, to which it is covalently linked through its amino group, in turn determines which of the three substituent bonds on C*α* of the substrate will be cleaved. In 1966, Dunathan [14] provided a unifying concept for the specific selection of the substrate scissile bond in PLP-dependent enzymes, which has been confirmed in solution and structural studies. In his proposal, all bonds broken and made on the catalytic pathway are the nearest perpendicular to the conjugated *π* system of the PLP ring. The resulting carbanion at C*α* of the substrate amino acid is stabilized by resonance with the *π*-electron system in the pyridine ring of PLP. This intermediate on the catalytic pathway is referred to as the “quinonoid” complex and absorbs near 500 nm (Scheme 1). Solution studies have shown that SHMT passes through several ordered, spectrophotometrically identifiable intermediates that reflect changes in the electron system of the PLP cofactor [12, 15] and are consistent with Dunathan's proposal.

 Prior to the availability of crystallographic structure information on SHMT, it was noted that the amino acid sequences of SHMTs from diverse species have a conserved run of 4 threonine residues terminating 2 residues upstream of the active site lysine: V-V-T-T-T^254^-T-H-K^257^(PLP)-T (numbering is that for rabbit cytosolic serine hydroxymethyltransferase (rcSHMT)) [16]. To determine the possible roles of this conserved sequence in SHMT catalysis, each threonine of this active site stretch was mutated in ecSHMT to an alanine, and the effects of the changes on the spectral and kinetic properties were investigated [17]. It was found that only the T226A mutant of ecSHMT (Thr226 in ecSHMT numbering is equivalent to rcSHMT Thr254) had significant spectral and kinetic differences from the wild type enzyme. There was a 32-fold lower *k*_cat_ in the conversion of L-serine to glycine, and the T226A mutant was virtually inactive toward cleavage of L-allo-threonine compared to wild type ecSHMT. Furthermore, in the presence of L-serine the T226A mutant exhibited a large spectral absorbance peak at 343 nm, which is characteristic of a *gem*-diamine intermediate and only a small peak at 425 nm characteristic of the external aldimine. Stopped-flow analysis showed that the 343 nm peak was formed rapidly, but its conversion to the 425 nm absorbing peak was slow [17]. Since the *gem*-diamine is generally a short-lived intermediate on the reaction pathways of PLP-dependent enzymes, this T226A ecSHMT mutant offered the opportunity to investigate the structural changes that apparently slowed the conversion of the *gem*-diamine intermediate to the external aldimine. We were unable to obtain crystals for the T226A mutant of *ec*SHMT. However, we were able to crystallize the homologous T254A and T254C mutants of rcSHMT. We report here the kinetic properties and crystal structures of the T254 mutants of rcSHMT and their glycine and L-serine complexes. In addition, we have increased the resolution of the structure of wild type rcSHMT to 2.1 Å as an aid in examining details of the *gem*-diamine structures.

## 2. Results and Discussion

### 2.1. Spectroscopic Studies

**Figure 1:**
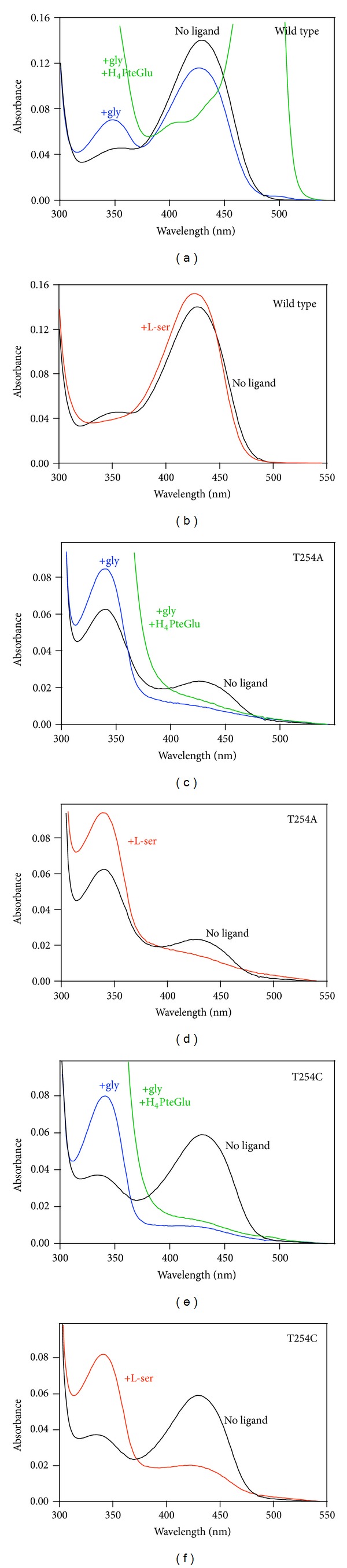
Absorption spectra of wild type and mutant rcSHMT in the absence and presence of substrates. Left panels show the absorption spectra of wild type, T254A and T254C rcSHMTs at 30°C before (black lines) and after the addition of 90% saturating glycine (blue lines). Green lines are spectra taken after the addition of 100 *μ*M H_4_PteGlu to the samples containing glycine. Right panels show the absorption spectra of the same enzymes before (black lines) and after the addition of 90% saturating L-serine (red lines).

 Wild type rcSHMT exhibits a characteristic major single absorption band with maximum intensity at 430 nm, due to the protonated internal aldimine between enzyme and cofactor. Also, a minor band is observed at 340 nm (Scheme 1). The addition of saturating concentrations of glycine results in a decrease of the major absorption band, with a slight blue shift at 426 nm and the appearance of a well-defined band at 343 nm. A small band centered at 498 nm is also visible. It is well established that these absorption bands correspond to the formation of the external aldimine, the *gem*-diamine, and the quinonoid intermediates, respectively [1, 12, 18]. All absorption bands of wild type and mutant enzymes are shown in Figure 1.

 Like the wild type, the T254A mutant form exhibits a 430 nm absorption band (although much less intense than wild type), indicative of the presence of an internal aldimine, and also shows an important band at around 340 nm that may represent either the enolimine form or the carbinolamine, a hydrated form of the internal aldimine in which the water molecule mimics the substrate nucleophilic attack and the *gem*-diamine formation. As shown in Figure 1, this band is usually present in very low concentration in the freshly purified wild type enzyme. The addition of a saturating concentration of glycine to T254A mutant results in the almost complete loss of absorbance at 430 nm, with a concomitant increase of absorbance at 343 nm. This effect was also observed with *E. coli* SHMT T226A mutant (residue Thr226 of *ec*SHMT corresponds to residue Thr254 in rcSHMT). Kinetic studies on the latter mutant showed that the complex absorbing at 343 nm is formed in a bimolecular step providing strong evidence that it is indeed the *gem*-diamine intermediate [17]. The addition of H_4_PteGlu to glycine-saturated wild type SHMT results in the increase of the 498 nm absorbing band, corresponding to the quinonoid intermediate. The large increase in absorbance below 400 nm is the absorbance of the excess H_4_PteGlu. Instead, when the T254A mutant was saturated with glycine, even in the presence of H_4_PteGlu, it did not show any 498 nm absorbing band. The effects of the addition of a saturating concentration of L-serine to T254A rcSHMT mutant enzyme are similar to those observed for glycine, with a decrease of the 430 nm absorbing band and a concomitant increase of the 343 nm absorbing species. In contrast, when wild type rcSHMT is saturated with L-serine, there are a marked increase and a blue shift of the major absorbing band, centered now at 426 nm, but no absorbance is observed at around 340.

 The spectral features of the unliganded T224C mutant are largely similar to those of the wild type enzyme. However, the 340 nm band is slightly more intense. If compared to the T254A mutant, the relative intensities of the 430 nm and 340 nm bands are reversed. Spectral changes upon substrates addition are similar to the ones observed with the T254A mutant, except that a residual absorbance is shown in the 420–430 nm region.

### 2.2. Kinetics Studies

 The spectral properties of the rcSHMT T254A and T254C mutant enzymes described above suggest that the *gem*-diamine intermediate accumulates upon substrate addition. The purified mutant enzymes were tested for catalytic activity using L-serine and L-allo-threonine as substrates (Table 1). For L-serine, both mutant enzymes showed slightly increased *K*_*m*_ values (about 2-fold) when compared to wild type rcSHMT; *k*_cat_ values decreased 46-fold for the Thr to Ala mutant and 531-fold for the Thr to Cys mutant. Similarly, *K*_*m*_ values for L-allo-threonine were found to be only slightly higher than wild type (less than 2-fold for both mutants), whereas *k*_cat_ values showed a 9- to 29-fold decrease for T254A and T254C mutants, respectively.

**Figure 2:**
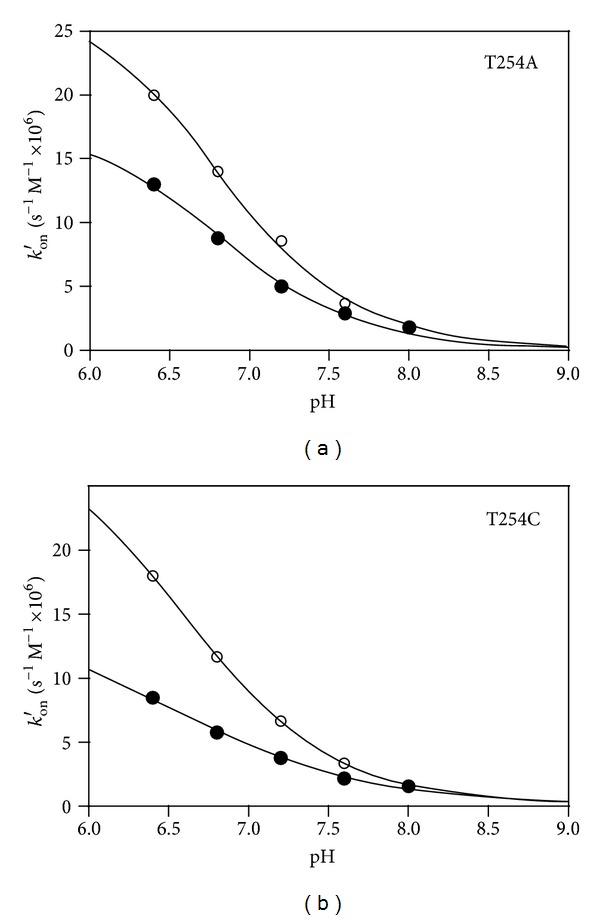
Dependence of *k*_*on*⁡_′ values determined from stopped-flow experiments on pH, for T254A and T254C rcSHMT mutants. Open circles correspond to data obtained with L-serine, while closed circles correspond to data obtained with glycine. The continuous lines through the experimental points were obtained through a least square minimization process using the equation for a sigmoidal curve (the software used was GraphPad, Prism).

 To better understand the role of Thr254 in the formation and breakdown of the *gem*-diamine intermediate and thus on the transaldimination reaction, the rate of the spectral changes occurring when L-serine and glycine were added to the enzyme was determined at different pH values by means of stopped-flow measurements. The *k*_*on*⁡_ and *k*_*off*⁡_ values for the rate of formation and breakdown of the enzyme-substrate complex absorbing at 343 nm were determined from a plot of *k*_obs_ versus substrate concentration, after linear regression of data and extrapolation of the slope and intercept values (Table 2). For both mutants and for both substrates at each pH value, *k*_obs_ was a linear function of substrate concentration, as expected for a second-order reaction. The second-order rate constants for substrate addition and *gem*-diamine formation (*k*_*on*⁡_) were corrected for the concentration of the amino acid anionic form at each pH value and are listed as *k*_*on*⁡_′. The anionic form of the *α*-amino group is assumed to be the true substrate for the transaldimination reaction. In the pH range used in the experiments (6.4–8.0), the concentration of the anionic form of the amino acid substrates increases about 40 times. The *k*_*on*⁡_ values increased with pH and varied from 3.3 · 10^4^ to 10.7 · 10^4^ s^−1^ *·*M^−1^ when L-serine was used as substrate and from 0.8 · 10^4^ to about 4.4 · 10^4^ s^−1^ *·*M^−1^ when glycine was used as substrate; *k*_*on*⁡_ values were very similar for both T254 mutant forms. After correction for the concentration of anionic substrate, *k*_*on*⁡_′ values showed a 10-fold decrease as pH increased from 6.4 to 8.0 and ranged from 20 · 10^6^ to 1.8 · 10^6^ s^−1^ *·*M^−1^ with L-serine and from 13 · 10^6^ to 1.8 · 10^6^ s^−1^ *·*M^−1^ with glycine. The sigmoidal dependence of *k*_*on*⁡_′ on pH suggests the titration of a group at the active site involved in a general acid catalysis, with a pK_a_ around 6.8 (Figure 2). If the internal aldimine of the mutant enzymes is mostly in the carbinolamine form, as inferred from the absorption spectra shown in Figure 1, it must be dehydrated (through the protonation of the hydroxyl group followed by the elimination of water) in order to be converted into the ketoenamine that is able to react with the incoming amino group of the substrates. Importantly, we have observed that the ratio of the 428 nm and 340 nm bands changes with pH in a similar manner, with the ketoenamine form being favored at higher pH values (data not shown). A possible candidate for this acid catalysis is Tyr73, which is involved in a cation-*π* interaction with Arg263. It has been shown that Tyr residues involved in cation-*π* interactions may have their pK_a_ lowered by 1 to 3 pH units (the normal pK_a_ of tyrosine residue is about 9.5) [19]. Furthermore, this residue points toward the protein region where the transaldimination reaction takes place, and its phenolic hydroxyl is located in close proximity to C4′ (see Figure 6). When L-serine was used as substrate, *k*_*off*⁡_ values showed a decreasing trend as pH was increased and ranged from 10.3 to 3.1 s^−1^ for the T254A mutant form and from 39 to 23 s^−1^ for T254C. In contrast, when glycine was used as substrate, *k*_*off*⁡_ values slightly increased with pH, ranging from 1 to 2.9 s^−1^ for the T254A mutant form and from 5.1 to 7.0 s^−1^ for T254C. 

 Stopped-flow studies done many years ago at pH 7.3 for wild type rcSHMT [12] gave a comparable *k*_*on*⁡_ value to the values for the T254A and T254C mutants. On the other hand, the *k*_*off*⁡_ values determined with the mutant enzymes are about 2 orders of magnitude lower than the wild type. These data suggest that the mutations have not significantly affected the rate of formation of the *gem*-diamine intermediate but have a more significant effect on the rate of its breakdown.

If the rapid spectroscopic changes occurring when the enzyme was mixed with saturating concentration of substrates were followed for a longer period of time (up to 50 seconds), a slow first-order increase was observed at 426 nm. For the T254A mutant with saturating concentration of L-serine, the rate constant was determined to be 10 min^−1^. This value is in agreement with previous studies on the T226A mutant of *ec*SHMT [17]. It is interesting to notice that this rate constant is very close to the *k*_cat_ measured for both hydroxymethyltransferase and retro-aldol cleavage reactions catalyzed by this mutant. This shows that for the T254A mutant the conversion of the *gem*-diamine into the external aldimine intermediate has become the rate-limiting step.

The spectral and kinetic studies both strongly suggest that removing the hydroxymethyl group of Thr254 by replacing it with either an Ala or a Cys residue greatly slows the conversion of the *gem*-diamine intermediates to the external aldimine. This may be the result of either blocking the conversion of *gem*-diamine I to *gem*-diamine II or of slowing conversion of *gem*-diamine II to the external aldimine. Therefore, the slowing of the catalytic cycle could be explained by either the mutant *gem*-diamine complexes being in a more stable form (in an energy well) compared to the wild type enzyme or by an increased energy barrier to pass to the external aldimine. These two hypotheses are not mutually exclusive and could be applied at the same time.

### 2.3. Crystal Structures

We determined the crystal structures of the T254A and T254C mutant enzymes and the complex of each with glycine and L-serine, and the structure of wild type rcSHMT to a higher resolution than had previously been attained [20].  Table 3 lists the crystallographic and refinement data for the new rabbit cytosolic SHMT structures determined and described in this work. All structures except for the T254C-glycine complex were solved in space group P4_1_ with a tetramer of 4 independently determined subunits per asymmetric unit. The T254C-glycine complex was solved in space group P4_1_2_1_2 with a dimer per asymmetric unit. The oligomeric structure of rcSHMT consists of two tight dimers each made of identical monomers. These tight dimers are loosely associated to form a so-called dimer of dimers. Disordered density recurred in the insert segment around residue 272 and at the amino termini of all monomer subunits, as observed in previously determined SHMT structures, but these disordered residues are not close to the regions of the enzyme around the PLP and substrate binding site, which are the subject of this study.

#### 2.3.1. Differences in Structure of Unliganded Wild Type and T254 Mutants

Except for the small differences in the active site described herein, all structures are virtually identical (rmsd <0.4 Å in all cases). The interactions of the PLP cofactor with the protein in the wild type and T254 mutants are almost identical. The distance of the Asp228 carboxylate to N1 of the pyridine ring is unchanged in the mutants as are the noncovalent bond constraints on the phosphoryl group of the PLP tail and the coplanarity of the His148 imidazole with the PLP ring. The active sites of all subunits of the wild type have phosphate or MES buffer anions bound where the carboxylate moiety of the amino acid substrate binds in SHMT-substrate complexes (see next paragraph). In the T254A mutant, this site is occupied by phosphate ions in one dimer and by MES in the other dimer. In the T254C mutant this anionic site is occupied by water in three subunits and by an apparent phosphate in the fourth subunit (Table 4).

**Figure 3:**
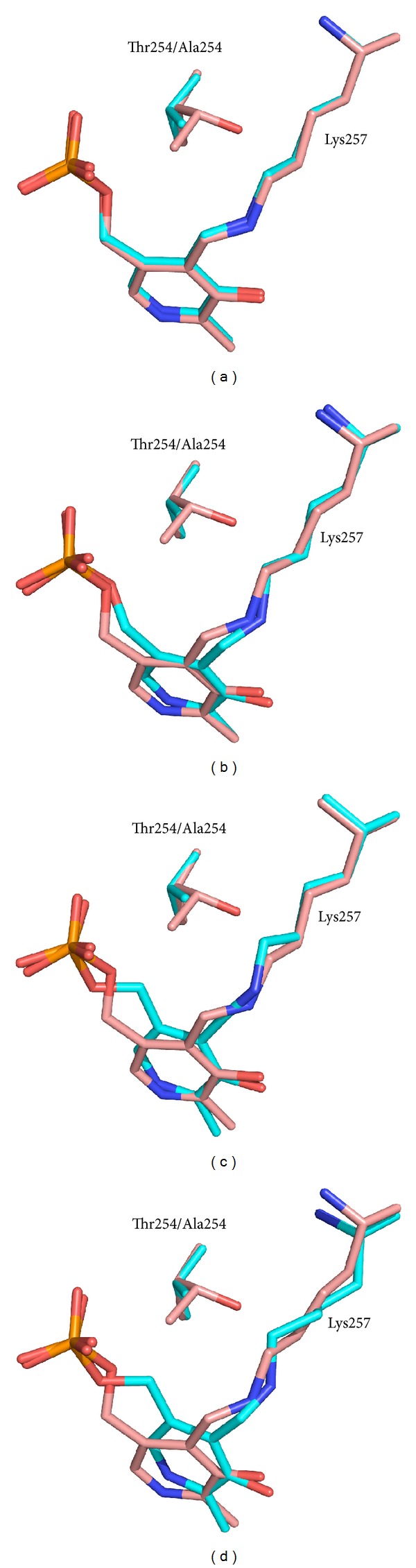
Superposition of the active site structures of rcSHMT wild type (salmon) and T254A (cyan) in the internal aldimine form. Panels (a) to (d) correspond to different subunits of the tetramer.

There are significant differences between the wild type and the T254 mutants in the conformation of the PLP methylene phosphate tail although these are seen to a different extent in different subunits (Figure 3). These differences correlate with the absence of the C*β* methyl group in the Ala254 and Cys254 side chains in the mutants. In the wild type structure, this methyl group of Thr254 projects toward the methylene phosphate tail. Replacement of the side chain by Ala or Cys opens a small space that allows the cofactor tail to assume a different conformation, moving the C5′ towards this cavity. This is the most evident change observed in the mutants. The fact that not all of the T254A and C subunits show this change to the same extent suggests that there is a low barrier to this switch of conformations and that the crystallization process is selecting out an asymmetric distribution. As a consequence of the elbow-like rotation of the methylene phosphate PLP tail in the mutant T254 forms, the PLP ring orientation also diverges from that of the wild type. Moreover, in the mutant enzyme structures with the largest PLP tail conformational differences, the aldimine linkage between C4′ and the *ε*-amino group of the active site lysine (Lys257) is far from being in the plane of the PLP ring (Figure 3(d)), as required by the double bond conjugation present in the ketoenamine form of the internal aldimine. As the aldimine linkage is driven away from this plane, its conjugation with the *π*-electron system of the pyrimidine ring is diminished, and the maximum absorbance is shifted to lower wavelengths [21], as observed for the mutant enzymes in solution (Figure 1). As discussed above, we cannot exclude that the 340 nm absorbing band of the unliganded mutant enzymes may correspond to the hydrated form of the internal aldimine (carbinolamine; Scheme 1). In the internal aldimine form of the T254C mutant, the conformational change of the methylene phosphate tail and the displacement of the PLP ring are less evident (data not shown). This correlates with the observation that, in the absorption spectrum of this mutant in solution, the amount of the canonical 428 nm ketoenamine band is higher than in the T254A mutant.

#### 2.3.2. The Glycine and L-Serine Complexes of T254A and T254C Mutants

**Figure 4:**
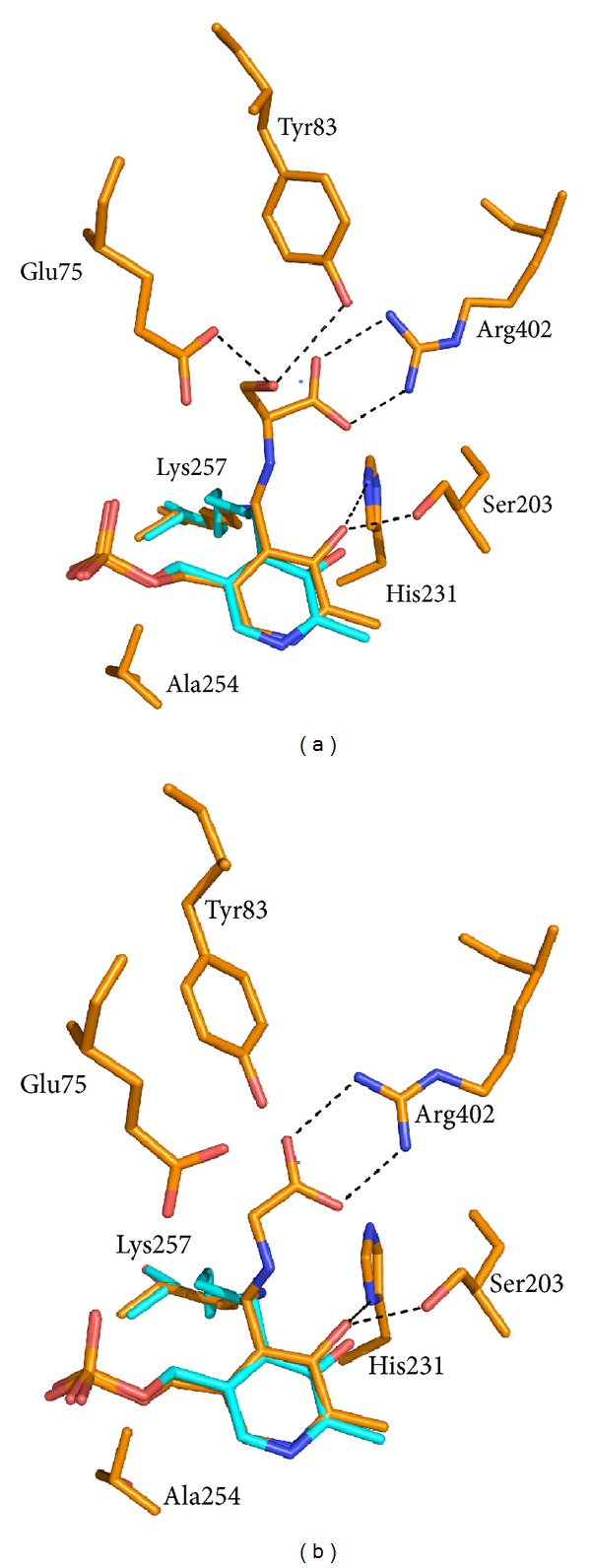
Superposition of the active site structures of the T254A mutant in the internal aldimine form (cyan) and as a *gem*-diamine complex (orange) with either L-serine (a) or glycine (b).

The structures of the T254A and T254C mutants with glycine and L-serine substrate ligands are all in the *gem*-diamine form (Table 4), showing both the amino group of the substrate and the *ε*-amino group of Lys257 forming bonds to C4′ of PLP. In all subunits of both mutants and with both substrates, the orientation of the PLP ring and the conformation of the methylene phosphate tail are very similar. Interestingly, the inhomogeneity seen in the internal aldimine structures of the mutant enzymes has disappeared upon binding of substrates. For example, Figure 4 shows a comparison between the active site structures of the T254A mutant with either L-serine (a) or glycine (b) bound to C4′ of PLP in the *gem*-diamine form and the internal aldimine. The comparison is made with the subunit of the unliganded mutant enzyme in which the internal aldimine shows the largest difference with respect to wild type (Figure 3(d)). It can be seen that, in both *gem*-diamine structures, the orientation of the PLP ring and the conformation of the methylene phosphate tail and of the active site lysine (Lys257) are very similar to those of the internal aldimine form and therefore differ significantly from the wild type internal aldimine structure. The carboxylate group of substrates is oriented to form dual hydrogen bonds with Arg402 and also with Y83. Residues Ser203 and His231 make hydrogen bonds with the O3′ of PLP, as already observed in all other SHMT structures. In the L-serine *gem*-diamine complex, the hydroxyl group of the substrate makes H-bond interactions with Glu75 and Tyr83, as also observed in the crystal structure of the *Bacillus stearothermophilus* SHMT-L-serine complex [22].

Wild type rcSHMT enzyme cocrystallized with glycine and 5-CHO-H_4_PteGlu showed two of the enzyme subunits in the *gem*-diamine form [19] (Table 4). No external aldimines were present, as the other two subunits were in the internal aldimine form. It is worth noting that structural variation among subunits in ligand binding site occupancy and type of intermediate complex is also found in other eukaryotic and prokaryotic SHMTs [22–26]. The above wild type rcSHMT-glycine-5-CHO-H_4_PteGlu ternary complex structure and that of mouse cytosolic SHMT (which was also cocrystallized with glycine and 5-CHO-H_4_PteGlu [25]) are the only wild type SHMT structures in which *gem*-diamine intermediates can be seen.

**Figure 5:**
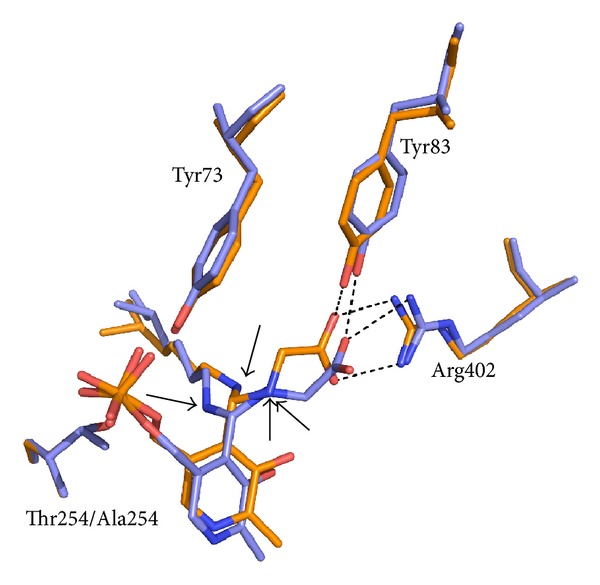
Superposition of the active site structures of the wild type (slate) and T254A mutant form (orange) of rcSHMT as *gem*-diamine complexes with glycine (pdb 1ls3). The arrows in the figure point towards the amino groups of the *gem*-diamine complexes.

When wild type and T254A rcSHMT-glycine *gem*-diamine structures are superimposed, some striking differences can be noticed (Figure 5). The methylene phosphate tail conformation and the orientation of the PLP ring are significantly different. The carboxylate group of glycine in wild type rcSHMT *gem*-diamine, although still interacting with Y83 and Arg402 through one of its oxygen atoms, is no more oriented to interact optimally with Arg402, and the other oxygen points away from it. Moreover, the position of the two amino groups in the *gem*-diamine is different between wild type and mutant (see arrows in Figure 5). In particular, the hydrogen atoms of the amino groups point away from Tyr73, which might be involved in the proton exchange required to interconvert the two *gem-*diamine intermediates (see below for discussion).

**Figure 6:**
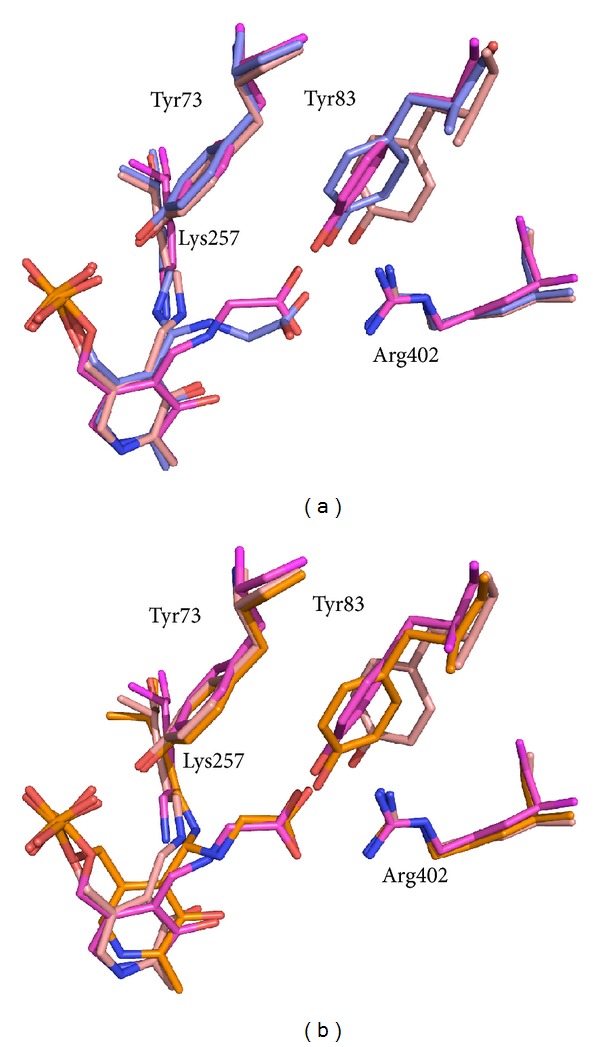
Superposition of wild type structures corresponding to the internal aldimine (salmon; rcSHMT) and external aldimine (magenta; mcSHMT, pdb 1eji) forms of the cofactor with the glycine *gem*-diamine form of the wild type (slate) and T254A mutant (orange).

In the wild type *gem*-diamine, the bond between the substrate amino group and C4′ of PLP lies roughly perpendicular to the cofactor ring, indicating a strong similarity to *gem*-diamine I intermediate, in which the amino group of the substrate has attacked the C4′ Schiff base of the cofactor with a trajectory that is perpendicular to the pyridine ring (Scheme 1). It appears that the wild type rcSHMT in the crystal has been trapped as a *gem*-diamine I intermediate that barely accumulates in solution. On the other hand, the *gem*-diamine forms found in T254 mutants seem to be in an aberrant position. The best way to appreciate this is to superimpose the structures of wild type *gem*-diamine (Figure 6(a)) or T254 mutant *gem*-diamine (Figure 6(b)) with wild type internal and external aldimines. In SHMTs, as PLP reacts with the substrate and the internal aldimine is converted into the external aldimine, the pyridine ring rotates by about 25°, primarily around the C2-C5 axis. This is also observed in prokaryotic *B. stearothermophilus* SHMT with both glycine and L-serine as substrates and in the ternary complex with glycine and 5-CHO-H_4_PteGlu [22]. In the wild type rcSHMT *gem*-diamine, the PLP ring lies between the positions observed in the internal and external aldimines. Strikingly, in the mutant T254 *gem*-diamine the cofactor ring has a different orientation, which is unlikely to correspond to that occurring in the transaldimination reaction. This is obviously a consequence of the mutation, since this position of the PLP ring is also observed in the unliganded forms of T254A and T254C rcSHMT structures. The *gem*-diamine structures of the mutant enzyme appear to be close to the *gem*-diamine II intermediate (Scheme 1), and the angle of the bond from C4′ of the PLP ring to the *ε*-amino group of K257 being close to the 90° predicted for this intermediate II, in which the amino group of Lys257 has to be eliminated in order to form the external aldimine intermediate. Moreover, the carboxylate group of the substrate makes optimal dual hydrogen bonds with Arg402 (Figure 6(b); structure in orange), as is observed in the external aldimine form of wild type SHMT-glycine complex (Figure 6(b); structure in magenta).

These observations suggest that in the T254 rcSHMT crystals, the PLP-substrate complex is blocked in the form of an anomalous and stable gem-diamine intermediate. As mentioned above, it is noteworthy that in the T254 mutant *gem*-diamine intermediates the position and orientation of the two amino groups point away from Tyr73 compared to the wild type *gem*-diamine structure. Tyrosine 73 was shown in *E. coli* and *bs*SHMT to have an important role in the transaldimination process and was proposed to act as proton exchanger between *gem*-diamines I and II. Interestingly, Tyr to Phe mutants of this residue also accumulate the *gem*-diamine intermediate [18, 27].

An additional remarkable variation in the T254 mutant structures compared to wild type is observed in the peptide bond between His256 and Lys257. Among the determined mutant structures, this peptide bond adopts two distinct orientations, one similar to the wild type rcSHMT and the other differing by up to 180°. The local variation in conformation of the His256-Lys257 peptide bond may be linked to the rcSHMT T254 mutations, since, in the internal aldimine of wild type rcSHMT, the carbonyl oxygen of His256, which is flipped in some mutant structures, makes a hydrogen bond through a water molecule to the Thr254 side chain. The lack of uniformity in structure among the subunits of the T254 mutants in the conformation of the PLP methylene phosphate tail, the position of the PLP ring, and the orientation of the His256-Lys257 peptide bond suggest that mutation of T254 relaxes some local structural constraints around the active site.

## 3. Conclusions

 The T254A and T254C mutations have created a small empty space in the active site of rcSHMT due to the absence of the threonine methyl group. This has allowed the methylene phosphate tail of PLP to adopt a stable, uncharacteristic conformation that is in turn responsible for an aberrant positioning of the PLP ring. This clearly affects the transaldimination step of the SHMT reaction, making it the rate-limiting step in the catalytic cycle. In these mutants, when either L-serine or glycine is added to the enzyme, *gem*-diamine intermediates greatly accumulate. This may be the result of either blocking the conversion of *gem*-diamine I to *gem*-diamine II, slowing the conversion of *gem*-diamine II to the external aldimine, or both.

 Conversion of the *gem*-diamine II into the external aldimine requires that the *ε*-amino group of Lys257 is protonated and that it leaves perpendicularly from the *si* face of C4′ of PLP. In Scheme 1 this is shown as a direct proton transfer from the substrate amino group in *gem*-diamine I to the leaving amino group of Lys257 in *gem*-diamine II. However, this proton transfer almost certainly does not occur directly but through shifts of protons in a network of acid-base groups at the active site. It is possible, based on the wild type and T254 mutant structures, that protonation of the Lys257 amine of the *gem*-diamine is mediated by Tyr73. The position of the *gem*-diamine amino groups relative to the Tyr73 side chain varies in the mutants. These local structure changes in T254 mutants could be responsible for a perturbation of the proton transfer chain which slows the transaldimination reaction.

 Available external aldimine SHMT structures (such as, *E. coli* SHMT as ternary complex with glycine and 5-CHO-H_4_PteGlu (pdb 1dfo) and *bs*SHMT with glycine and L-serine (pdb 1kl1 and 1kkp, resp.)) all show a good hydrogen bond between the Lys257 *ε*-amino group and the hydroxyl group of the Thr254 side chain, as originally suggested by Pascarella et al. [16]. The nucleophilicity of the *ε*-amino group and the NH_2_ of the substrate must be balanced so that transaldimination can occur rapidly from either direction. The hydrogen bond between Thr254 and the Lys257 *ε*-amino group is likely to be a critical determinant of this balance. In the T254A and T254C mutants, which lack the hydroxyl group, this H-bond cannot be formed. We suggest that the loss of this hydrogen bond destabilizes the external aldimine of the T254 mutants relative to the *gem*-diamine intermediates and results in the trapping of the latter.

 PLP-dependent enzymes typically catalyze the reversible transaldimination reaction between the internal and external aldimines very rapidly, within the dead time of stopped-flow measurements, and do not accumulate *gem*-diamine intermediates. This is a crucial step in the catalytic mechanism of all PLP-dependent enzymes because it determines binding of substrates and release of products. Our studies show that a single, semiconservative mutation of an active site residue can be critical for the transaldimination in SHMT.

## 4. Materials and Methods

### 4.1. Materials

All chemicals, coenzymes, antibiotics, and buffers were from Sigma-Aldrich (St. Louis, MO, USA) or FisherScientific (Pittsburgh, PA, USA). (6S)-H_4_PteGlu and (6S)-5-CHO-H_4_PteGlu were gifts from Merck Eprova AG (Schaffhausen, Switzerland). Crystallization buffers were from Hampton Research (Laguna Niguel, CA, USA).

### 4.2. Mutagenesis, Expression, and Purification of SHMT

Mutants were made using the QuikChange site-directed mutagenesis kit from Stratagene (La Jolla, CA, USA) on rcSHMT cDNA in the pET22b vector [28]. The T254A and T254C mutant forms were produced using the primers 5′-CGTGGTGACCACCGCGACCCACAAGACGC-3′ and 5′-CGTGGTGACCACCTGCACCCACAAGACGC-3′, respectively, and their complementary oligonucleotides (the mutated codons are underlined). Each mutation was confirmed by sequencing the cDNA insert in both directions. Oligonucleotides synthesis and DNA sequencing were performed by Eurofins MWG Operon (Ebersberg, Germany). Each mutant protein was expressed in an *E. coli* HMS174(*λ*DE3), and purification was done by the same procedure published previously [23] and resulted in high yields of >95% pure enzymes that exhibited the size of wild type rcSHMT.

### 4.3. Spectra and Kinetic Studies

All spectra and steady state kinetic studies were performed in a cell with a path length of 1 cm with an Agilent 8354 spectrophotometer at 30°C, in a 20 mM potassium phosphate buffer, pH 7.3, containing 5 mM 2-mercaptoethanol and 0.2 mM ethylenediaminetetraacetic acid. Kinetic assays were performed as previously reported [23]. Briefly, catalytic assay for L-serine and H_4_PteGlu was measured by coupling the product CH_2_-H_4_PteGlu to methylenetetrahydrofolate dehydrogenase with the concomitant reduction of NADP to NADPH. To determine the *K*_*m*_ for L-serine, H_4_PteGlu was maintained at 0.15 mM, and the L-serine concentration was varied between 0.05 mM and 5 mM. The concentration of mutant rcSHMT in these assays was 10 *μ*M. The rate of L-allo-threonine cleavage was assayed by coupling the reduction of the product acetaldehyde with NADH and alcohol dehydrogenase. Kinetic constants values were determined from double-reciprocal plots of the decrease in absorbance at 340 nm with L-allo-threonine concentrations varied between 0,5 and 50 mM.

### 4.4. Rapid Reaction Studies

Stopped-flow absorbance experiments were performed with an Applied Photophysics SX18 apparatus (Leatherhead, UK) equipped with a 1 cm optical path observation chamber. Temperatures were held at either 8°C by a circulating water bath. Each study was an average of 4–6 traces.

The rate of formation of the *gem*-diamine intermediate was measured by following the increase at 343 nm for the first 0.4 seconds after mixing enzyme and substrate solutions. The effect of substrate concentration was determined by varying L-serine and glycine concentrations in a 0.5–5 mM range. The curves for absorbance variation versus time were fit by a single-exponential curve. For each concentration of substrate, both the first-order rate constant, *k*_obs_, and the amplitude of the spectra change were determined. Rate constants and amplitudes for each individual reaction varied less than 10% from the average values for each reaction:

For calculation of *k*_*on*⁡_ and *k*_*off*⁡_ the following equation was applied, assuming the reaction to be pseudo-first toward [*E*], and [*S*] equal to free substrate concentration:

(1) $$\begin{array}{c} k_{\text{obs}}=k_{\mathrm{on}}\left[S\right]{+k}_{\mathrm{off}}. \end{array}$$

The values of *k*_*on*⁡_ and *k*_*off*⁡_ for the rate of formation and breakdown of the enzyme-substrate complex absorbing at 343 nm can be then determined from the slope and *y*-axis intercept of the *k*_obs_ versus [*S*] graph. A double-reciprocal plot of absorbance changes at 343 nm versus substrate concentration gives a linear fit with an *x*-axis intercept representing *K*_*d*_. The enzyme concentration was held constant at 40 *μ*M. The buffer used was a mix of 20 mM potassium 2-(N-morpholino) ethanesulfonic acid (MES), 20 mM N,N-bis[2-hydroxyethyl]-2-aminoethane sulfonate (BES), and 20 mM 4-(2-hydroxyethyl)-1-piperazineethanesulfonic acid (HEPES) brought to pH 6.4, 6.8, 7.2, 7.6, and 8.0. The concentrations of the anionic forms of amino acids were calculated using the Henderson-Hasselbalch equation and pK values of 9.6 and 9.2 for the amino groups of glycine and L-serine, respectively.

### 4.5. Crystallization

All forms of the rcSHMT (50 mg/mL) and its mutants and complexes were crystallized as previously reported [20] from 2-3% PEG4000, 20 mM K_2_HPO_4_/KH_2_PO_4_, and 50 mM potassium MES or sodium HEPES (pH 7.0) in hanging drops or in 0.5 mL Eppendorf tubes at room temperature. The complexes with glycine and serine had 60 mM of the amino acid in the crystallization drop.

#### 4.5.1. Data Collection and Structure Determination

 Crystals were transferred to a stabilization solution of 4.8% polyethylene glycol 4000 in the same buffer for 1 hour, then transiently (<30 sec) placed in a cryoprotectant of 30% polyethylene glycol 400 and 6% polyethylene glycol 4000 in 50 mM potassium MES (pH 7.0) and flash-frozen in liquid N_2_ for data collection. Data for a 100° sector were collected on a RAxisII with Osmics confocal optics at 60 kV and 150 mA. Oscillation frames were integrated with Denzo and merged with Scalepack [29]. Merged intensity data were converted to structure factor amplitudes using Truncate refmac5 [30]. All crystals except the T254C-glycine were indexed in space group P4_1_ with a tetramer per asymmetric unit; T254C-glycine was indexed in space group P4_1_2_1_2 with one dimer per asymmetric unit. Structures were solved by molecular replacement using a rabbit cytosolic SHMT dimer from pdb entry 1CJ0 as a search model and refined using alternating cycles of manual fitting into SigmaA weighted 2mF_o_dF_c_ maps in COOT [31] and computational refinement in CNS [32] and refmac5 [30]. Structure factors and coordinates are being deposited to the RCSB protein databank.

## Figures and Tables

**Table 1:** Kinetic constants for the hydroxymethyltransferase and retro-aldol cleavage reactions catalyzed by wild type and T254 mutant rcSHMT forms.

Substrate	Kinetic constant	Form of rabbit cytosolic SHMT		
		Wild type	T253A	T253C
L-Serine	*K*_*m*_ (mM)	0.3	0.6	0.5
	*k*_cat_ (min^−1^)	850	18.5	1.6

L-allo-Threonine	*K*_*m*_ (mM)	1.5	2.5	2.6
	*k*_cat_ (min^−1^)	130	14.3	4.5

**Table 2:** Kinetic constants for the rate of formation and breakdown of the *gem*-diamine complex for mutant rcSHMT forms with glycine and L-serine substrates.

Substrate	pH	T253A			T253C		
		*k*_*on*⁡_ (s^−1^ M^−1^ · 10^4^)	*k*_*on*⁡_′ (s^−1^ M^−1^ · 10^6^)	*k*_*off*⁡_ (s^−1^)	*k*_*on*⁡_ (s^−1^ M^−1^ · 10^4^)	*k*_*on*⁡_′ (s^−1^ M^−1^ · 10^6^)	*k*_*off*⁡_ (s^−1^)
L-Serine	6.4	3.3	20	10.3	2.9	18	39
	6.8	5.8	14	8.3	4.0	10	36
	7.2	8.7	8.6	5.8	6.8	6.7	32
	7.6	9.5	3.7	5.3	8.7	3.4	27
	8.0	10.7	1.8	3.1	9.4	1.5	23

Glycine	6.4	0.8	13	1.0	0.5	8.5	5.1
	6.8	1.3	8.0	1.3	0.7	4.5	6.7
	7.2	2.1	5.0	1.9	1.5	3.8	6.9
	7.6	3.0	2.9	2.4	2.1	2.2	7.0
	8.0	4.4	1.8	2.9	3.7	1.6	6.8

**Table 3:** Crystallographic data collection and refinement statistics for rcSHMT structures determined in this work.

	Wild type	T254A	T254A + gly.	T254A + L-ser.	T254C	T254C + gly.	T254C + L-ser.
Cell/space group	115.6,115.6,156.0 P4_1_	115.1,115.1,157.4 P4_1_	115.0,115.0,156.8 P4_1_	115.5,115.5,156.3 P4_1_	114.0,114.0,154.7 P4_1_	114.6,114.6,156.9 P4_1_2_1_2	114.0,114.0,155.6 P4_1_
Resolution	119.0–2.10 (2.16–2.10)	111.8–2.55 (2.62–2.55)	20.0–2.65 (2.72–2.65)	115–2.40 (2.46–2.40)	111.8–2.40 (2.46–2.40)	90.0–2.65 (2.72–2.65)	111.8–2.55 (2.62–2.55)
Completeness (%)	98.5 (98.2)	92.2 (87.9)	99.6 (99.8)	88.3 (85.8)	90.1 (86.9)	69.6 (62.0)	90.9 (76.9)
*R*_work_	.199 (.248)	.201 (.306)	.203 (.292)	.197 (.247)	.195 (.242)	.221 (.333)	.202 (.284)
*R*_free_	.242 (.316)	.271 (.432)	.277 (.399)	.258 (.318)	.256 (.346)	.305 (.414)	.263 (.362)
*N*_work_	105631 (7668)	55295 (3849)	52905 (3769)	63363 (4478)	62364 (4393)	19976 (1244)	52787 (5963)
*N*_free_	11736 (845)	6204 (426)	5968 (406)	7156 (510)	7009 (470)	1654 (96)	5963 (375)
〈*B*_protein_〉	30.8	44.8	34	37.3	39.4	36.6	38
〈*B*_water_〉	39.8	43.8	34.6	37.3	41.9	NA	40.1
RMSD from ideal							
Bond lengths	0.008	0.007	0.007	0.006	0.006	0.006	0.007
Bond angles	1.3	1.24	1.22	1.13	1.13	1.08	1.16
Ramachandran plot							
Favored	1460	1424	1403	1425	1435	702	1427
Additionally allowed	123	142	142	146	133	69	128
Generously allowed	13	8	9	12	15	6	12
Forbidden	3	7	9	5	5	7	10
MolProbity score	1.73 (93%)	2.04 (96%)	2.04 (97%)	1.88 (96%)	1.81 (97%)	2.24 (93%)	2.00 (96%)

**Table 4:** Rabbit cytosolic SHMT structures compared in this study. The table shows the cofactor form and the ligand present in each subunit of the structures.

Enzyme	Chain	Form	Ligand
Unliganded wild type	A	i.a.	PO_4_^2−^
	B	i.a.	PO_4_^2−^
	C	i.a.	MES
	D	i.a.	MES

Unliganded T254A	A	i.a.	PO_4_^2−^
	B	i.a.	PO_4_^2−^
	C	i.a.	PO_4_^2−^
	D	i.a.	PO_4_^2−^

T254A + glycine	A	g.d.	Gly.
	B	g.d.	Gly.
	C	g.d.	Gly.
	D	g.d.	Gly.

T254A + L-serine	A	g.d.	L-Ser.
	B	g.d.	L-Ser.
	C	g.d.	L-Ser.
	D	g.d.	L-Ser.

Unliganded T254C	A	i.a.	H_2_O
	B	i.a.	H_2_O
	C	i.a.	H_2_O
	D	i.a.	PO_4_

T254C + glycine	A	g.d.	Gly.
	B	g.d.	Gly.

T254C + L-serine	A	g.d.	L-Ser.
	B	g.d.	L-Ser.
	C	g.d.	L-Ser.
	D	g.d.	L-Ser.

Wild type + glycine + 5-CHO-H_4_PteGlu_3_	A	g.d.	Gly.
	B	i.a.	—
	C	g.d.	Gly.
	D	i.a.	—

i.a.: internal aldimine.

g.d.: *gem*-diamine.

MES: 2-(N-morpholino)ethanesulfonate.

5-CHO-H_4_PteGlu_3_: triglutamic form of 5-formyltetrahydrofolate.

## Abbreviations

SHMT: Serine hydroxymethyltransferase
rc: Rabbit cytosolic
mc: Mouse cytosolic
ec: *Escherichia coli*
bs: *Bacillus stearothermophilus*
PLP: Pyridoxal 5′-phosphate
*gem*-diamine: Geminal diamine
H_4_PteGlu: Tetrahydrofolate (tetrahydropteroylglutamate)
5,10-CH_2_-H_4_PteGlu: 5,10-Methylenetetrahydrofolate
5,10-CH^+^=H_4_PteGlu: 5,10-Methenyltetrahydrofolate
5-CHO-H_4_PteGlu: 5-Formyltetrahydrofolate.
